# Timing and predictors of disease incidence among named contacts of reported tuberculosis patients in a low incidence setting

**DOI:** 10.1371/journal.pone.0313801

**Published:** 2025-05-07

**Authors:** Michael Asare-Baah, Lori Johnston, Lina Dominique, Michael Lauzardo, Marie Nancy Séraphin

**Affiliations:** 1 Emerging Pathogens Institute, University of Florida, Gainesville, Florida, United States of America; 2 Division of Infectious Diseases and Global Medicine, University of Florida, College of Medicine, Gainesville, Florida, United States of America; 3 Florida Department of Health, Bureau of Tuberculosis Control, Tallahassee, Florida, United States of America; London School of Hygiene and Tropical Medicine, UNITED KINGDOM OF GREAT BRITAIN AND NORTHERN IRELAND

## Abstract

Contact investigations are crucial for tuberculosis (TB) control, yet the temporal dynamics of disease progression among exposed contacts remain poorly understood. We conducted a retrospective cohort study of 44,106 contacts linked to 6,243 index TB cases diagnosed between 2009 and 2023. During the 15-year follow-up, 454 contacts developed TB disease, with 43.4% being incident cases. Using time-to-event analysis with left truncation to account for varying follow-up times, and mixed effect Cox models to account for index case and county-level variability, we estimated the median time to TB incidence at 11 (IQR 4–48) months after initiating contact investigation. The risk of TB disease varied markedly by age and immune status. Children aged 0–15 showed over nine times higher risk compared to adults aged 25–44 (aHR = 9.59; 95% CI: 3.17–29.02; p < 0.001). Contacts co-infected with HIV demonstrated a three-fold increased risk of TB (aHR = 2.35; 95% CI: 1.08–5.10; p = 0.031) relative to those without HIV. A history of a previous TB diagnosis conferred a protective effect on the risk of TB incident (aHR = 0.40; 95% CI: 0.20–0.80; p = 0.009). Additionally, individuals who had incomplete therapy for latent TB infection (LTBI) also experienced a protective effect (aHR = 0.32; 95% CI: 0.15–0.71; p = 0.005). These findings highlight a critical window for intervention with follow-up needed for at least 1–4 years after initial contact investigations. The results also emphasize the need for targeted, risk-stratified surveillance and LTBI treatment for children and individuals with HIV who are contacts of confirmed TB cases.

## Introduction

Tuberculosis (TB) remains a formidable global health challenge, affecting not just those initially infected but also their close contacts. Contact’s vulnerability stems from their heightened exposure to *Mycobacterium tuberculosis* (MTB), the causative agent, transmitted via airborne droplets [1]. Close contacts of pulmonary TB cases represent a high-risk group for disease development and onward transmission of MTB [2], and as such are key targets for preventive measures [3].

Contact investigations remain a crucial strategy for controlling TB among contacts, especially in low-incidence settings [4]. These investigations allow for the identification of both TB disease and latent TB infection (LTBI) among exposed contacts, facilitating prompt treatment and thereby interrupting further transmission [5]. Early detection and treatment of contacts with LTBI are critical for preventing the progression to TB disease, while rapid identification and treatment of TB cases minimize the ongoing spread [3]. In our settings, TB contact investigations are completed within three working days of identifying an index case. High-priority contacts are examined for TB infection within seven working days and receive a medical assessment within 10 working days [6]. This process typically involves a comprehensive approach, including clinical assessments, chest radiography, microbiological evaluation of sputum, and tests for LTBI such as tuberculin skin tests (TST) or interferon-γ release assays (IGRA) [7,8].

Factors, including the infectiousness of the index case, proximity and duration of exposure, and the inherent susceptibility of the contact influence the risk of infection [7,9,10]. The incidence of new TB cases among contacts following infection is not uniform over time. It is markedly elevated during the first year post-exposure, gradually declining but remaining persistently above baseline for at least five years [7,11,12]. Several factors have been identified as increasing the risk of TB disease progression among contacts. These include younger age (especially children under 5), older age (individuals over 65) [13,14], HIV infection, inadequate antiretroviral therapy in HIV-positive individuals, a history of prior TB treatment, low socioeconomic status, certain ethnicities, comorbidities such as diabetes, and specific host genetic polymorphisms [15]. Health and lifestyle factors such as poor nutritional status, smoking, and alcohol consumption have also been associated with elevated risk of TB among contacts [16]. Contacts with LTBI who did not receive or complete latent LTBI treatment are also likely to develop TB [17].

Understanding the temporal dynamics and risk factors associated with TB progression among contacts is crucial for developing targeted and effective public health interventions. In identifying those at the highest risk and the critical time window for intervention, resources can be more efficiently allocated, and preventive measures can be tailored to maximize impact. This knowledge gap forms the rationale for our study, which aims to investigate the time to TB disease onset and the specific risk factors influencing disease incidence among contacts of confirmed TB cases.

## Methods

### Study design and data source

We conducted a retrospective cohort study using two primary data sources obtained from the Florida Department of Health (FDOH) Bureau of TB Control. The TB case dataset captures demographic, clinical, and epidemiological data for all reported TB cases, as documented in the Report of a Verified Case of Tuberculosis (RVCT) form [18]. The TB contact tracing dataset contains comprehensive information on contacts identified, screened, and diagnosed with LTBI or TB disease. These two datasets collected for TB disease surveillance and clinical management of cases were linked using unique identifiers to create a dataset of index TB cases reported between 2009–2023 and their contacts, which was used for this analysis. Data extraction and linkage were conducted on August 20, 2024, following institutional review board (IRB) approvals.

### Human ethics approval and consent to participate statement

This study received approval from the Institutional Review Board (IRB) at the University of Florida (approval number IRB201700445). It was conducted as part of program evaluations and projects to improve the clinical and public health services offered by state and local TB programs in Florida. The approval authorities waived the requirement for informed consent, as the research used existing records and did not involve direct interaction with participants.

### Study population and selection criteria

In our setting, exposed contacts of confirmed TB cases are defined as individuals sharing the same airspace with an infectious individual for a significant period, often quantified as more than 15 hours per week for one or more weeks or accumulating over 180 hours during the infectious period [12]. These contacts may reside in the same household, share social settings, or be linked through workplace or school environments. Our study population consisted of 44,106 individuals identified and investigated as contacts of 6,243 culture-confirmed TB cases reported between January 1, 2009, and December 31, 2023. Among these contacts, 454 individuals were reported as TB cases during the follow-up period and were included in our analysis. Our analysis did not consider the risk of TB progression among contacts diagnosed with LTBI as all contacts were recorded as not having LTBI. Nonetheless, it included contacts who received LTBI therapy but did not complete their treatment.

### Measures

#### Outcome definition.

The primary outcome of interest was the development of TB disease among contacts, categorized as either incident or co-prevalent cases. We defined incident cases as contacts diagnosed with TB disease more than 30 days post-initiation of contact investigation [12]. Co-prevalent cases were defined as contacts diagnosed with TB disease before or within 30 days of initiating contact investigation [12]. TB disease diagnosis was confirmed based on positive culture results for MTB. The 30-day threshold was chosen based on previous epidemiological studies conducted in the US and Canada [12] with TB epidemiology similar to Florida and accounts for the typical time required for diagnostic procedures and reporting.

#### Follow-up.

We defined the time to disease onset as the number of months between the start date of the contact investigation for the index case and the earliest date the contact was either diagnosed with or reported as having TB. The follow-up period for each contact began on the date of the initial contact investigation for the index case. The endpoint was the date of diagnosis for contacts who developed TB disease or the end of the study follow-up (December 31, 2023) for those who remained disease-free.

#### Predictor variables.

We examined a comprehensive set of risk factors for TB incidence among contacts, categorized into demographic, clinical, and social variables. Demographic factors included: age (0–15, 16–24, 25–44, 45–64, and ≥ 65 years), sex (male/female), race (White, Black, Asian), ethnicity (Hispanic/non-Hispanic), and birth origin (U.S.-born/non-U.S.-born). Clinical factors comprised co-infection with HIV status, diabetes, non-HIV immunosuppression (cancers, chronic renal or liver diseases), incomplete LTBI therapy, and previous TB diagnosis. Social variables included smoking status at diagnosis (ever smoker, never smoker, unknown), self-reported recreational drug use, alcohol use, and homelessness within the past year. Additional variables assessed were missed contact status, contact relationship to the index case (immediate family, coworker, social contact, other), and estimated contact duration (≤1 day, 2–14 days, 15–30 days, > 30 days).

### Statistical analysis

To assess differences in sample characteristics between incident and co-prevalent cases, we employed Pearson’s chi-square test for categorical variables and Student’s t-test or Mann-Whitney U test for continuous variables, depending on their distribution using the stats package in R (version 4.3.3)[19].

We calculated annual age-standardized TB incidence rates per 100 person-years. This was done by dividing the number of incident TB cases by the total person-years at risk and standardizing using annual age-stratified TB rate data obtained from Florida Charts [20] The age standardization was performed using the epitools package (version 0.5–10.1) in R [21], which implements the direct method of standardization [22].

We used Kaplan-Meier product limit estimates to estimate the probability of TB disease-free survival over the follow-up period. These estimates were calculated using the survival package (version 3.5–5) in R [23]. We plotted the resulting Kaplan-Meier curves stratified by key risk factors using the survminer package (version 0.4.9) [24]. We compare the survival distributions between categories of covariates using log-rank tests [25]. We report p-values from these tests to indicate statistical significance of differences between survival distributions.

We fitted bivariable and multivariable mixed-effect Cox models with left truncation to assess the individual and combined effects of the selected risk factors on TB incidence. These models incorporated Gaussian random effects using a Laplace approximation to account for the complex structure of our data [26]. Our models included two levels of random effects: A random intercept at the index case patient level to account for unobserved heterogeneity and inherent clustering in TB transmission data. The significance of this random effect was assessed using a chi-square test (χ² = 149.43, p = 3.91e-11) and county-level random effects (χ² = 73.05, p = 4.08e-06) to account for geographical variability in contact investigation practices. This multi-level approach allowed us to capture both individual-level and spatial heterogeneity in TB transmission risk.

We rigorously assessed the proportional hazards assumption using global tests based on Schoenfeld residuals. This assessment was conducted for each covariate independently and for the overall multivariable model. A Schoenfeld residuals chi-square test p-value < 0.05 indicated a failure to reject the null hypothesis that the residuals are randomly distributed over time, suggesting no violation of the proportional hazards assumption [27]. The results of these tests are presented in S1 Fig, which includes plots of scaled Schoenfeld residuals against transformed time for each covariate, allowing for visual inspection of the proportional hazards assumption [26]. We present bivariable and multivariate hazard ratios (HRs) with 95% confidence intervals (CIs) for the disease-free survival probability associated with each predictor variable. All statistical analyses were conducted in R version 4.3.3 (R Core Team, 2024) [28]. A two-sided α of 0.05 or less was considered statistically significant.

## Results

### Characteristics of contacts who developed TB disease (Table 1)

Among 454 TB contacts who developed the disease, significant differences emerged between co-prevalent (n = 257) and incident (n = 197) cases. Age distribution varied significantly (p = 0.005), with co-prevalent cases being more prevalent in the 25–44 age group (38.1% vs. 29.9%), while incident cases were more prevalent in the 45–64 age group (40.1% vs. 31.5%). Non-US birth origin was more common among co-prevalent cases (45.1% versus 29.9%, p = 0.001), consistent with differences in racial distribution (p = 0.031), where co-prevalent cases showed higher proportions of Asian (9.4% versus 3.6%) and Black (43.0% versus 40.8%) contacts.

**Table 1:** Characteristics of contacts who developed TB disease stratified by co-prevalent and incident cases, Florida, 2009–2023.

Study Variables	Study Sample 454 (%)	Co-prevalent TB Disease 257 (%)	Incident TB Disease 197 (%)	P - value
Age groups in years (%)				0.005
0-15	35 (7.7)	28 (10.9)	7 (3.6)	
16-24	66 (14.5)	31 (12.1)	35 (17.8)	
25-44	157 (34.6)	98 (38.1)	59 (29.9)	
45-64	160 (35.2)	81 (31.5)	79 (40.1)	
65+	36 (7.9)	19 (7.4)	17 (8.6)	
Sex = Male (%)	268 (59.0)	151 (58.8)	117 (59.4)	0.968
Race (%)				0.031
Asian	31 (6.9)	24 (9.4)	7 (3.6)	
Black	190 (42.0)	110 (43.0)	80 (40.8)	
White	231 (51.1)	122 (47.7)	109 (55.6)	
Hispanic = Yes (%)	123 (27.1)	77 (30.0)	46 (23.4)	0.143
Non-US-Born = Yes (%)	175 (38.5)	116 (45.1)	59 (29.9)	0.001
Contacts Co-infected with HIV (%)				0.475
No	374 (82.4)	207 (80.5)	167 (84.8)	
Yes	46 (10.1)	28 (10.9)	18 (9.1)	
Unknown	34 (7.5)	22 (8.6)	12 (6.1)	
Diabetic = Yes (%)	40 (8.8)	20 (7.8)	20 (10.2)	0.474
Non-HIV Immunosuppression = Yes (%)	12 (2.6)	5 (1.9)	7 (3.6)	0.445
Previous TB Diagnosis = Yes (%)	25 (5.5)	8 (3.1)	17 (8.6)	0.019
Past Year Recreational Drug Use = Yes (%)	80 (17.6)	33 (12.8)	47 (23.9)	0.003
Past Year Alcohol Use = Yes (%)	116 (25.6)	56 (21.8)	60 (30.5)	0.047
Smoking Status at Diagnosis (%)				0.012
Never Smoker	23 (5.1)	11 (4.3)	12 (6.1)	
Ever Smoker	17 (3.7)	4 (1.6)	13 (6.6)	
Unknown/Missing	414 (91.2)	242 (94.2)	172 (87.3)	
Past Year Homelessness = Yes (%)	48 (10.6)	22 (8.6)	26 (13.2)	0.150
Missed Contact = Yes (%)	12 (2.6)	5 (1.9)	7 (3.6)	0.445
Incomplete LTBI = Yes (%)	14 (3.1)	4 (1.6)	10 (5.1)	0.061
Relationship with Index Case (%)				0.063
Immediate family	241 (53.1)	149 (58.0)	92 (46.7)	
Social Contact	89 (19.6)	44 (17.1)	45 (22.8)	
Coworker	16 (3.5)	6 (2.3)	10 (5.1)	
Other	108 (23.8)	58 (22.6)	50 (25.4)	
Duration of Exposure to Index Case				0.002
<= 1 day	18 (4.0)	8 (3.1)	10 (5.1)	
2-14 days	143 (31.5)	72 (28.0)	71 (36.0)	
15 -30 days	53 (11.7)	22 (8.6)	31 (15.7)	
>30 days	240 (52.9)	155 (60.3)	85 (43.1)	

Behavioral risk factors showed an inverse pattern, with incident cases demonstrating significantly higher rates of recreational drug use (23.9% versus 12.8%, p = 0.003), alcohol use (30.5% versus 21.8%, p = 0.047), and smoking status at diagnosis (4.6% versus 0.8%, p = 0.026). Previous TB diagnosis was more common among incident cases (8.6% versus 3.1%, p = 0.019). Exposure duration differed significantly (p = 0.002), with co-prevalent cases having longer contact periods (60.3% reporting >30 days versus 43.1% for incident cases). The groups showed no significant differences in sex, Hispanic ethnicity, co-infection with HIV status, diabetes, non-HIV immunosuppression, homelessness, missed contact, incomplete LTBI, or relationship to the index case.

### Timing of incident TB disease among contacts (Fig 1)

Panel A depicts the overall disease-free survival distribution. The median time to disease onset was 11 (IQR: 4–48) months. The probability of remaining disease-free drops rapidly within the first 12 months, stabilizing at approximately 50% at the median point, then continues to gradually decline over the follow-up period of 144 months.

**Fig 1:**
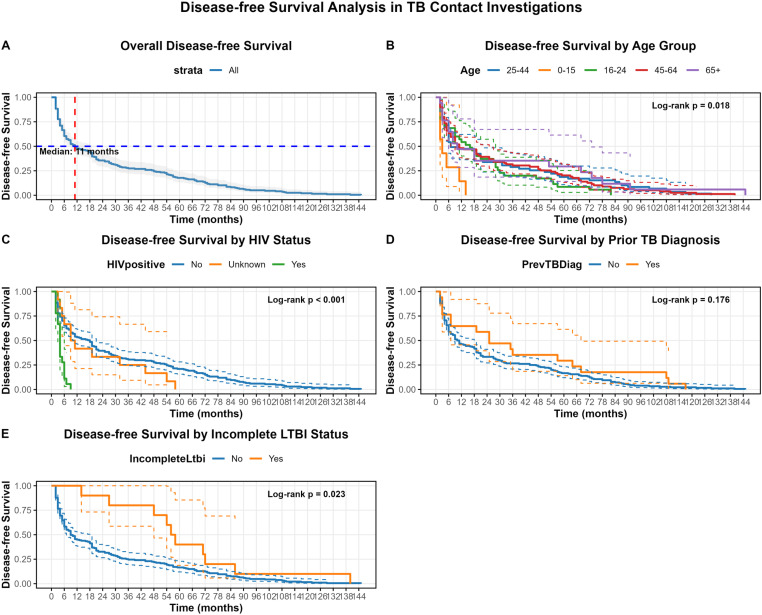
Disease-free survival curves for tuberculosis (TB) contacts. Overall distribution (Panel A; median time to disease onset = 11 months); Significant differences in disease-free survival observed across age categories (Panel B; log-rank test p = 0.018), co-infection with HIV (Panel C; log-rank test p < 0.001) and incomplete LTBI status (Panel E; log-rank test p = 0.023). There were no significant differences in disease-free survival for contacts with a history of previous TB diagnosis (Panel D: log-rank test p = 0.176) and those without.

Panel B illustrates disease-free survival stratified by five age groups. The log-rank test suggests significant differences in survival distributions between age groups (p = 0.018), with the 0–15-year group showing the most rapid decline in disease-free survival early in follow-up.

Panel C compares disease-free survival based on HIV status. The log-rank test indicates highly significant differences between groups (p < 0.001). HIV-positive individuals demonstrate markedly poorer disease-free survival, with a sharp decline within the first 12 months. The unknown HIV status group shows intermediate outcomes with wider confidence intervals, reflecting greater uncertainty in this subgroup.

Panel D presents disease-free survival stratified by a history of prior TB diagnosis. The log-rank test suggests that the difference between these groups is not statistically significant (p = 0.176). However, the graph indicates that individuals with a prior TB diagnosis tend to have slightly higher disease-free survival probabilities than those without a previous TB history.

Panel E compares disease-free survival between contacts who received incomplete LTBI therapy and those who received no LTBI therapy. The log-rank test reveals significant differences between the two groups (p = 0.023), with contacts who received incomplete LTBI therapy demonstrating a higher probability of disease-free survival.

### Risk factors associated with contacts developing incident TB disease (Table 2)

In our cohort of 197 individuals, younger age (0–15 years) was significantly associated with increased TB incidence risk in both bivariable (HR = 8.45; 95% CI: 2.70–26.37; p < 0.001) and multivariable (aHR = 9.59; 95% CI: 3.17–29.02; p < 0.001) analyses compared to adults aged 25–44 years. Contacts co-infected with HIV also showed an increased risk of TB in both bivariable (HR = 2.36; 95% CI: 1.13–4.93; p = 0.023) and multivariable (aHR = 2.35; 95% CI: 1.08–5.10; p = 0.031) models. Conversely, a history of previous TB diagnosis was protective against TB incidence in both bivariable (HR = 0.48; 95% CI: 0.24–0.96; p = 0.038) and multivariable analyses (aHR = 0.40; 95% CI: 0.20–0.80; p = 0.009). Additionally, incomplete latent TB infection (LTBI) therapy was associated with reduced TB risk in both bivariable (HR = 0.38; 95% CI: 0.16–0.90; p = 0.029) and multivariable analyses (aHR = 0.32; 95% CI: 0.15–0.71; p = 0.005).

**Table 2:** Risk factors associated with being an incident TB case among contacts.

Study Variables	Bivariate hazard ratio (HR) (95% CI)	P - value	Multivariate hazard ratio (HR) (95% CI)	P - value
Population at risk	197		197	
Number of events	197		196	
Age groups in years				
25-44	1.00		1.00	
0-15	8.45 (2.70, 26.37)	<0.001	9.59 (3.17, 29.02)	<0.001
16-24	1.51 (0.85, 2.71)	0.162	1.38 (0.79, 2.39)	0.255
45-64	1.16 (0.72, 1.88)	0.537	1.03 (0.64, 1.66)	0.895
65+	0.84 (0.40, 1.77)	0.641	0.64 (0.30, 1.36)	0.248
Sex = Male	0.93 (0.62, 1.40)	0.742	1.32 (0.86, 2.03)	0.198
Race				
White	1.00		1.00	
Black	1.35 (0.88, 2.07)	0.168	1.18 (0.69, 2.01)	0.543
Asian	0.62 (0.18, 2.10)	0.439	0.54 (0.17, 1.69)	0.287
Hispanic = Yes	1.04 (0.63, 1.71)	0.877	1.05 (0.54, 2.01)	0.894
Non-US-Born = Yes	0.92 (0.59, 1.43)	0.714	1.45 (0.86, 2.45)	0.162
Contacts Co-infected with HIV				
No	1.00		1.00	
Yes	2.36 (1.13, 4.93)	0.023	2.35 (1.08, 5.10)	0.031
Unknown	1.51 (0.68, 3.34)	0.311	1.57 (0.72, 3.44)	0.258
Diabetic = Yes (%)	0.64 (0.33, 1.23)	0.180	0.78 (0.39, 1.55)	0.479
Non-HIV Immunosuppression = Yes	0.63 (0.22, 1.83)	0.397	0.92 (0.34, 2.52)	0.878
Previous TB Diagnosis = Yes	0.48 (0.24, 0.96)	0.038	0.40 (0.20, 0.80)	0.009
Past Year Recreational Drug Use = Yes	0.92 (0.58, 1.46)	0.715	1.03 (0.63, 1.68)	0.914
Past Year Alcohol Use = Yes	0.80 (0.52, 1.25)	0.329	0.83 (0.51, 1.36)	0.456
Past Year Homelessness = Yes	1.48 (0.82, 2.65)	0.192	1.51 (0.81, 2.79)	0.194
Missed Contact = Yes	1.47 (0.52, 4.16)	0.464	1.39 (0.52, 3.72)	0.514
Incomplete LTBI = Yes	0.38 (0.16, 0.90)	0.029	0.32 (0.15, 0.71)	0.005
Relationship with Index Case				
Immediate family	1.00		1.00	
Social Contact	0.69 (0.41, 1.18)	0.174	0.80 (0.49, 1.31)	0.378
Coworker	1.60 (0.60, 4.29)	0.349	1.80 (0.70, 4.65)	0.225
Other	1.18 (0.71, 1.96)	0.524	1.22 (0.77, 1.92)	0.395
Duration of Exposure to Index Case				
<= 1 day	1.00		1.00	
2-14 days	0.57 (0.20, 1.61)	0.289	0.67 (0.26, 1.71)	0.402
15 -30 days	0.77 (0.26, 2.31)	0.639	0.73 (0.27, 1.99)	0.544
>30 days	0.72 (0.26, 2.02)	0.533	0.86 (0.34, 2.15)	0.746

**Notes:** Cox analysis excludes smoking status due to the high number of missing data points

## Discussion

In this statewide cohort study utilizing 15 years of data, we investigated the timing and risk factors associated with TB disease incidence among contacts of confirmed TB cases in Florida. The TB incidence trend in our study population ranged from 0.138 to 1.719 cases per 100 person-years (S2 Fig), which is lower than in other settings, where it ranges from approximately 1.13 to 2.36 cases per 100 person-years [29–31]. This suggests a low transmission rate, and/or more effective preventive measures in our population. Variations in incidence rates across low-incidence regions can be attributed to heterogeneity in study populations, methodologies, local TB control strategies, population demographics, exposure risks, underlying health determinants, and the prevalence of multidrug-resistant TB.

Our analysis reveals distinct patterns in disease onset and identifies key demographic and clinical factors that influence TB risk. The median time to TB disease onset of 11 (IQR: 4–48) months post-initiation of contact investigations observed in our study represents a critical window for surveillance and intervention. This finding aligns with previous studies that have documented similar temporal patterns in TB disease progression among contacts of infectious index cases [5,7,32–34]. The extended timeframe observed in the incidence of TB among contacts has important implications for TB control programs. First, it reveals potential delays in diagnosis and treatment that may contribute to sustained transmission risks [35,36]. Second, it demonstrates that contact susceptibility to TB infection and disease progression persists well beyond initial exposure, necessitating prolonged monitoring strategies. While the concentration of cases within the first year should guide resource allocation for TB control programs, our findings suggest that surveillance cannot be limited to the immediate post-exposure period. Extended follow-up, particularly for high-risk contacts, remains crucial for capturing late-onset cases and preventing subsequent transmission. Currently, TB contact investigations are completed within three working days of identifying an index case, with high-priority contacts examined for TB within seven working days and receiving a medical assessment within 10 working days [6]. These results emphasize the need to maintain active surveillance for at least 1–4 years after initial contact investigation to effectively identify and treat incident TB cases.

Our findings revealed distinct demographic and clinical features associated with TB disease risk among contacts, with age and HIV status emerging as critical determinants. Children aged 0–15 years demonstrated significantly higher susceptibility to TB compared to the 25–44 year group, consistent with previous studies [7,37,38]. This heightened risk likely reflects their immature immunity, which is less capable of containing TB infections compared to young adults [39,40]. The rapid manifestation of TB within 90 days post-exposure, particularly in young children [13], underscores the acute vulnerability of this demographic group and the urgency for targeted preventive interventions. Interestingly, older adults (≥65 years) demonstrated lower relative risk compared to children, possibly due to broader acquired immunity and different exposure patterns. However, this finding should be interpreted cautiously, considering the limited sample size for these age groups in our study and potential confounders such as the presence of comorbidities that could modify TB susceptibility. The absence of significant associations for other traditional risk factors suggests that TB progression dynamics among contacts may differ from those in the general population.

HIV co-infection emerged as another major risk factor for TB disease progression. HIV infection significantly weakens the immune system, particularly by reducing CD4 + T-cell counts, which are crucial for fighting infections like TB [41]. This immunosuppression makes individuals more susceptible to TB, whether through reactivation of latent TB or new infection [42]. The increased risk underscores the importance of regular TB screening in individuals with HIV and vice versa.

The unexpected protective effect of a previous TB diagnosis on the risk of developing TB among contacts can be attributed to several factors. Individuals with previous TB infection may have developed a robust immune response that provides partial protection against subsequent infections [43,44]. This immune memory could reduce the likelihood of progressing to active TB disease upon re-exposure [43,44]. Moreover, these individuals are likely to be more vigilant about symptoms and seek medical attention promptly, leading to early detection and treatment of re-infections [7]. It is also plausible that those with a history of TB diagnosis might have received some form of treatment for LTBI, which could lower the risk of reactivation and subsequent disease [15] as evidenced by the protective effect of incomplete LTBI therapy. This finding suggests that even partial treatment of LTBI might reduce the risk of developing active TB compared to no treatment at all [17] and warrant further investigation to optimize prevention strategies.

Our findings are limited by the inherent challenges of using programmatic data, which is primarily collected for clinical management and surveillance rather than for research purposes. Key limitations include issues such as missing data and unmeasured confounders. Important factors, such as nutritional status, the presence of cavitary lesions, and the smear positivity status of index cases, had either a high proportion of missing values or were unavailable in the dataset. Hence, the results should be interpreted with caution, as these important confounders were not considered. Future investigations will benefit from addressing this knowledge gap.

Our findings provide critical insights for TB control strategies, highlighting the urgent need to prioritize vulnerable populations. Specifically, we recommend enhanced surveillance and targeted LTBI treatment for young children and individuals with HIV who have been in contact with a confirmed TB case.

## Supporting information

S1 Fig Schoenfeld Residual Plots for Verification of the Proportional Hazards Assumption in Cox Regression Analysis. The plots display Schoenfeld residuals against time for each covariate in our Cox proportional hazards model. Horizontal trend lines with minimal deviation indicate that the proportional hazards assumption is satisfied for the respective covariates. Statistical tests for non-zero slopes are provided alongside each plot to quantitatively assess violations of this critical assumption.
(TIF)

S2 Fig Temporal Trend of TB disease incidence among exposed contacts from 2009-2023. (TIF)
