# Supplementary figures and images for: Timing and predictors of disease incidence among named contacts of reported tuberculosis patients in a low incidence setting
Source: PLoS One. 2025 May 7;20(5):e0313801. doi: 10.1371/journal.pone.0313801 (PMC12058168; doi:10.1371/journal.pone.0313801)

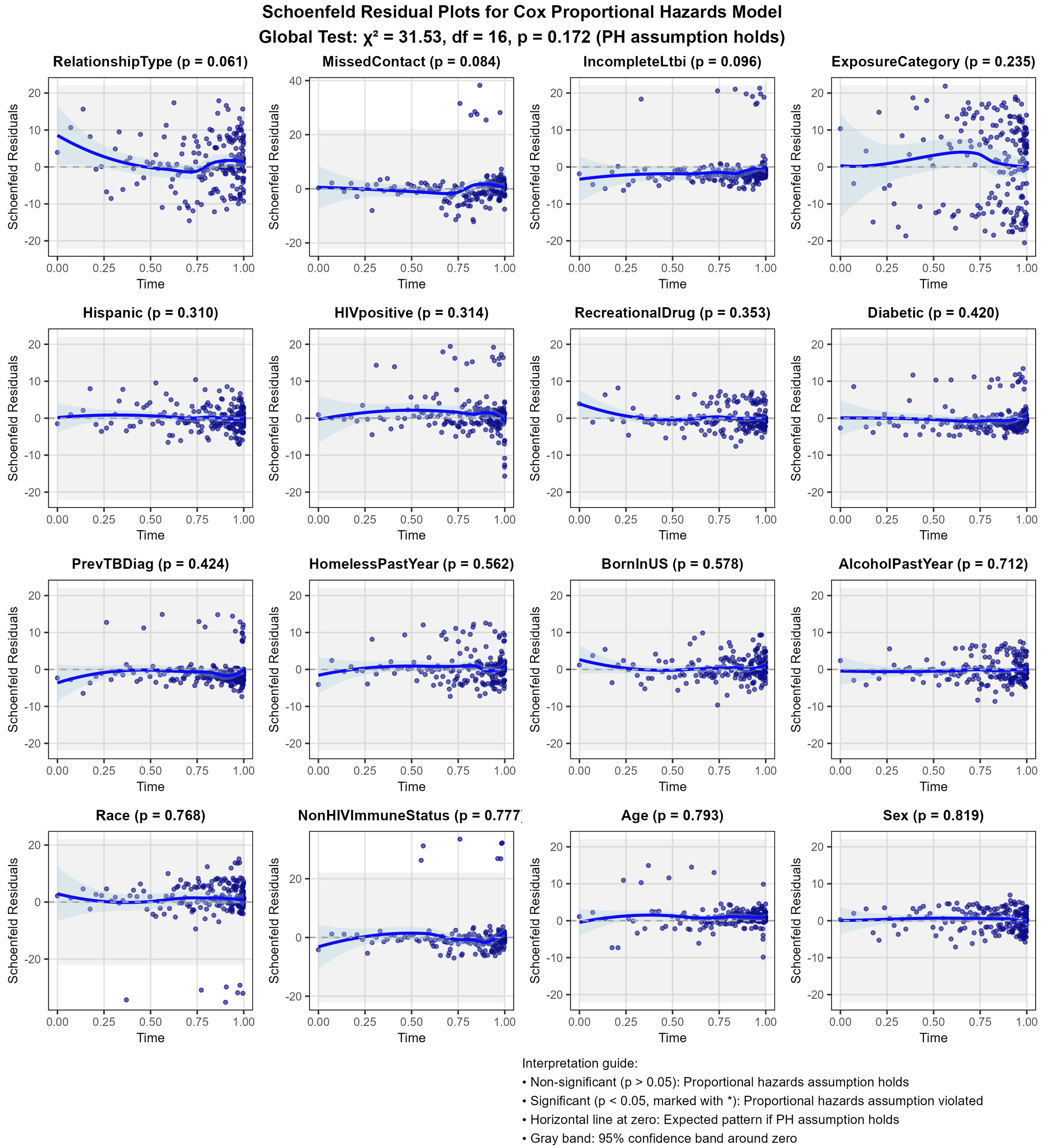

Supplement: S1 Fig — The plots display Schoenfeld residuals against time for each covariate in our Cox proportional hazards model. Horizontal trend lines with minimal deviation indicate that the proportional hazards assumption is satisfied for the respective covariates. Statistical tests for non-zero slopes are provided alongside each plot to quantitatively assess violations of this critical assumption. (TIF) [file pone.0313801.s001.tif]

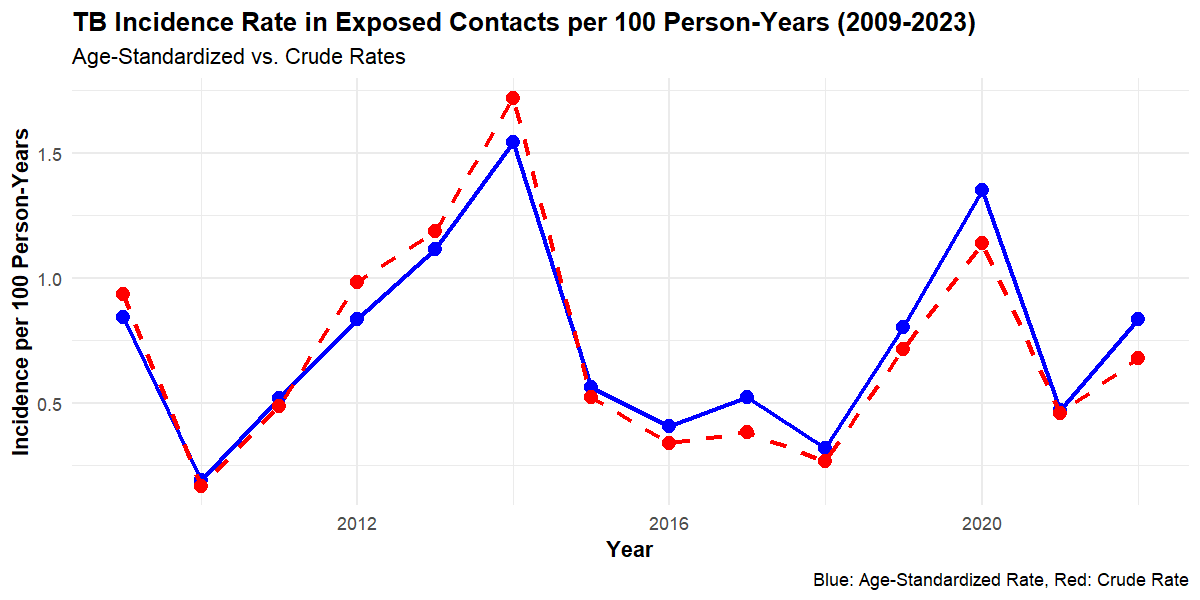

Supplement: S2 Fig — (TIF) [file pone.0313801.s002.tif]
